# Controlled Rejuvenation of Amorphous Metals with Thermal Processing

**DOI:** 10.1038/srep10545

**Published:** 2015-05-26

**Authors:** Masato Wakeda, Junji Saida, Ju Li, Shigenobu Ogata

**Affiliations:** 1 Graduate School of Engineering Science, Osaka University, 1-3 Machikaneyama, Toyonaka, Osaka, 560-8531, Japan; 2 Frontier Research Institute for Interdisciplinary Sciences, Tohoku University, Aramaki aza Aoba 6-3, Aoba-ku, Sendai, Miyagi, 980-8578, Japan; 3 Department of Nuclear Science and Engineering and Department of Materials Science and Engineering, Massachusetts Institute of Technology, 77 Massachusetts Avenue, Cambridge, Massachusetts, 02139, USA; 4 Center for Elements Strategy Initiative for Structural Materials (ESISM), Yoshida Honmachi, Sakyo-ku, Kyoto University, Kyoto, 606-8501, Japan

## Abstract

Rejuvenation is the configurational excitation of amorphous materials and is one of the more promising approaches for improving the deformability of amorphous metals that usually exhibit macroscopic brittle fracture modes. Here, we propose a method to control the level of rejuvenation through systematic thermal processing and clarify the crucial feasibility conditions by means of molecular dynamics simulations of annealing and quenching. We also experimentally demonstrate rejuvenation level control in Zr_55_Al_10_Ni_5_Cu_30_ bulk metallic glass. Our local heat-treatment recipe (rising temperature above 1.1*T*_g_, followed by a temperature quench rate exceeding the previous) opens avenue to modifying the glass properties after it has been cast and processed into near component shape, where a higher local cooling rate may be afforded by for example transient laser heating, adding spatial control and great flexibility to the processing.

Amorphous metals^1^,^2^,^3^ have excellent properties such as high corrosion resistance^4^, high strength^5^, and large elastic elongation^6^. At room temperature, however, plastic deformation localizes into narrow band region, and sudden fracture occurs exhibiting almost no macroscopic plastic deformation under uniaxial tensile and compressive loadings^7^,^8^,^9^. This brittle nature is the most serious flaw of amorphous metals for use as structural materials. Therefore, improvement, and moreover tuning of the deformability has been the largest challenge of the decades in the field of amorphous metals^10^,^11^,^12^,^13^. Rejuvenation^14^,^15^,^16^,^17^ is the structural excitation of amorphous solids accompanied by an increase in the enthalpy and free volume and is thus the inverse of aging. Since rejuvenation has to change the elastic and plastic deformation behavior^17^ of amorphous metals, it is a new but promising approach for tuning the deformability of amorphous metals. It has recently been reported that shot-peening^16^ and sever plastic deformation^17^ can mechanically induce the rejuvenation. On the other hand, structural excitation driven by a thermal loading process^18^,^19^ also has potential application for practical usage, because the thermal loading process realizes a local rejuvenation control, which is more difficult by mechanical processing. However, the feasibility rejuvenation driven by thermal annealing and quenching is still controversial because of few experimental evidence^18^ and a lack of clear knowledge of the nonequilibrium glass properties.

In this work, we theoretically propose a recipe to control the level of rejuvenation through systematic thermal processing and clarify the crucial feasibility conditions by means of molecular dynamics (MD) simulations and figure out a rejuvenation map with respect to annealing temperature and quenching rate. Our rejuvenation map provides temperature-cooling rate conditions to achieve desired level of rejuvenation while avoiding crystallization and/or gross shape change. Based on the rejuvenation map we also experimentally demonstrate the realization of rejuvenation level control in Zr_55_Al_10_Ni_5_Cu_30_ bulk metallic glass, which evidences our rejuvenation map and recipe do work well even for typical experimental cooling rates. Moreover, the internal structural features and mechanical properties of rejuvenated amorphous metals are revealed.

## Simulation Procedures

**Figure 1:**
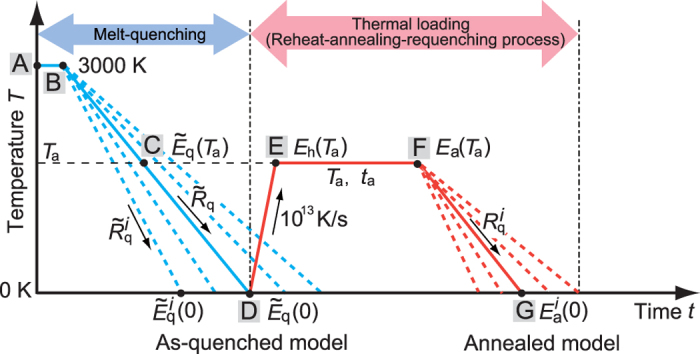
Schematic illustration of the initial melt-quenching and subsequent thermal loading process. The system was initially quenched at a constant cooling rate of 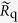 (B
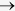
D). The instantaneous potential energy at temperature 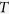 in the initial-quenching process is represented as 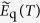. The as-quenched model was then subjected to thermal loading consisting of heating (D
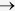
E), isothermal annealing (E
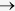
F), and quenching (F
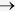
G) at various annealing temperatures 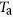 and constant cooling rates 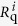. The instantaneous potential energy at temperature 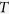 in the second quenching process (F
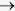
G) is represented as 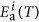.

For all of the MD simulations described here, we use the Lennard-Jones (L-J) potentials^20^ developed for the Cu-Zr amorphous alloy and a 30,000-atom simulation box with three-dimensional (3D) periodic boundary conditions. We consider alloys with atomic compositions of Cu_50_Zr_50_, Cu_30_Zr_70_, and Cu_57_Zr_43_ to ensure composition-independent generality (see Supplementary Information in regard to the latter two). The MD time step was set to 1 fs, and we employed the *NPT* ensemble for which the temperature and pressure are controlled by the Nose-Hoover^21^ and Parrinello-Rahman methods^22^, respectively. An amorphous model was constructed via a melt-quenching process as follows (Fig. 1). We first arranged the atoms randomly in the simulation box and melted the structure by keeping the model at a high temperature of 3000 K for 20 ps (Fig. 1; process A
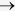
B). The melted model was then quenched at a constant cooling rate of 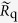
(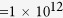 K/s) from 3000 K to 0 K (process B
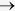
D). This quenched product is referred to as the “as-quenched model” hereafter, and we use a tilde to represent the initial quenching process. By monitorinng the specific volume change during the rapid quenching process with a cooling rate of 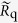, the glass transition temperature 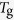 was determined to be approximately 900 K from the kink in volume-temperature curve. Since 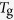 generally depends on the cooling rate^23^, we also determined a set of temperatures 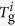 for five different cooling rates 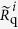 (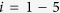): 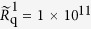 K/s, 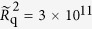 K/s, 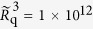 K/s, 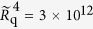 K/s, and 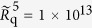 K/s (see Supplementary Information, Fig. S1).

We conducted successive thermal loading simulations of the as-quenched model as follows. First, the as-quenched model was reheated at a heating rate of 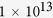 K/s up to a temperature 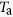 (process D
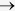
E) and then annealed for a time period 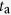 (=2000 ps) under constant 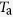 and zero external pressure conditions (process E
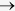
F). Isothermal annealing processes with 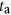
=500 and 1000 ps were also conducted and revealed that the annealing time 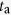 had a small effect on our simulation results (see Supplemental Information, Fig. S2). We assumed various 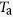 values ranging from 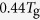 to 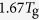 to examine the effect of the annealing temperature on the level of thermal rejuvenation. After isothermal annealing at 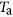 for 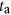, the model was quenched again at five different cooling rates, 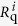 (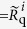) (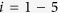), from 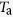 to 0 K (process F
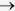
G). This final product is referred to as the “annealed model” hereafter.

## Results and Discussion

### Rejuvenation map

**Figure 2:**
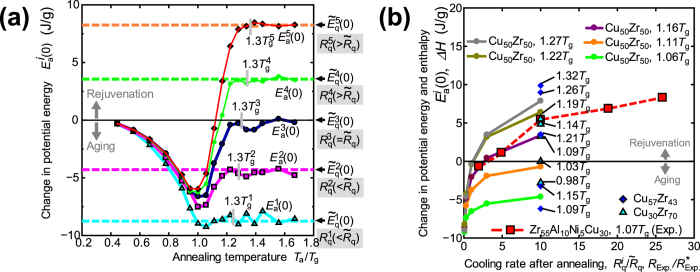
Rejuvenation map. (**a)** Change in the potential energy caused by the thermal loading process (D
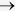
G), where 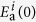 represents the potential energy of the annealed model with a cooling rate of 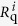. The potential energy of the as-quenched model with a cooling rate 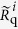 is shown by the dashed lines. The grey rectangles represent 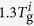, where 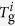 is the estimated glass transition temperature in the melt-quenching process with a cooling rate 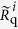
(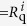). (**b**) Change in the potential energy calculated by MD and the change in the enthalpy obtained from the experiment. For the cooling rate after annealing, 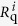 is normalized by 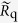 in the MD simulations, and 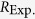 is normalized by *R*^∗^_Exp._ for the experimental data.

Figure 2(a) shows the generated excess potential energy 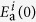 due to the thermal loading process from state D to G for different 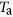 and 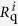 values. If any aging occurs during the thermal loading process, then the excess potential energy value should fall below zero. On the other hand, if thermal rejuvenation occurs, then the excess potential energy value should remain positive. Thus, the excess potential energy can represent the level of rejuvenation or aging depending on the sign. As we see in Fig. 2(a), significant aging is observed whenever the cooling rate is less than 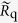, regardless of the annealing temperature 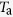, whereas thermal rejuvenation is observed in the opposite case. Thus, a higher cooling rate after isothermal annealing than that of the initial quenching process is a *necessary* condition for thermal rejuvenation, but this alone is insufficient. Thermal rejuvenation is only realized when the annealing temperature is above a certain critical temperature 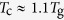. Thus, the two conditions, 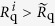 and 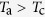, are crucial conditions for feasible thermal rejuvenation. We also found that a higher 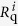 and a higher 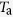 result in a higher level of rejuvenation under these conditions. Figure 2(a) can then be used as a rejuvenation map to identify conditions for controlling the rejuvenation level. It should be noted that 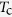 is not an intrinsic material property but depends on both 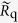 and 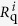 as follows: 1) Since a higher 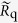 leads to a thermodynamically unstable as-quenched state, further aging proceeds easily during later thermal loading, and the 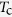 value is shifted to a higher temperature. 2) Since a higher 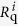 does not allow a more thermodynamically stable state to be found during the final quenching process, limited aging proceeds, and the 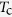 value is shifted to a lower temperature (Fig. 2(a)).

The levels of aging and rejuvenation are relative quantities and depend on the choice of reference amorphous state. Thus, if we choose a perfectly rejuvenated amorphous state as the reference amorphous state, which is supposedly constructed through melt quenching at an infinitely fast cooling rate, then further rejuvenation will not occur. In reality, however, it is impossible to realize such a perfectly rejuvenated state; even though a near-perfectly rejuvenated state can be realized, we would not be able to observe it because of the extremely short lifetime of the highly excited state. For this reason, the discussions above are valid for actual glass systems in general, in which both aging and rejuvenation can be realized.

It is worth noting that evaluating the level of aging and rejuvenation based on the volume change (i.e., mass density change) yields qualitatively similar results to those in Fig. 2(a) (see Supplementary Information, Fig. S3).

Based on the above findings, we demonstrate thermal rejuvenation experimentally in a Zr_55_Al_10_Ni_5_Cu_30_ bulk glassy alloy (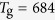 K at a heating rate of 0.33 K/s). The as-quenched Zr_55_Al_10_Ni_5_Cu_30_ alloy rod was sliced into 0.5-mm-thick disc samples that were initially annealed at 685 K for 120 s at heating and cooling rates of 0.17 K/s, denoted as *R*^∗^_Exp_. The fully relaxed samples were then annealed again at 735 K (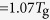) for 120 s at a heating rate of 0.33 K/s and then quenched at cooling rates of 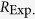 = 0.33, 0.83, 1.7, 3.2, or 4.4 K/s (Ref. 24). The excess enthalpy generated during the second annealing process 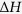 is calculated as 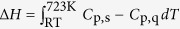, where RT is the room temperature. 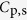 and 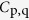 are the specific heats of the sample reheated to 723 K and the as-secondary annealed state, respectively. The specific heat measurement was conducted with a heating and cooling rates of 0.33 K/s using DSC. Figure 2(b) shows the change in the potential energy calculated by the MD simulation compared to the change in the enthalpy obtained from the experiment. The cooling rates after annealing employed in the MD simulation and experiment are normalized by the typical cooling rates, 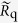 (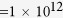 K/s) and *R*^∗^_Exp._ (=0.17 K/s), respectively. Note that due to the different alloy chemistries, the MD and the experimental curves in Fig. 2(b) are not directly comparable; yet the basic trend and the order of magnitude roughly match. The enthalpy obtained from the experiment increases with increasing cooling rate after annealing, and the positive enthalpy of the plots for 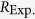
= 0.83, 1.7, 3.2, and 4.4 K/s indicate that thermal rejuvenation was achieved. This is clear evidence of the feasibility of thermal rejuvenation via thermal loading, provided the necessary conditions are satisfied. The rough matching between MD and experimental curves in Fig. 2(b) implies that the level of rejuvenation is dominated by a cooling rate ratio between initial melt-quenching and final quenching, rather than the absolute magnitude of the cooling rate.

**Figure 3:**
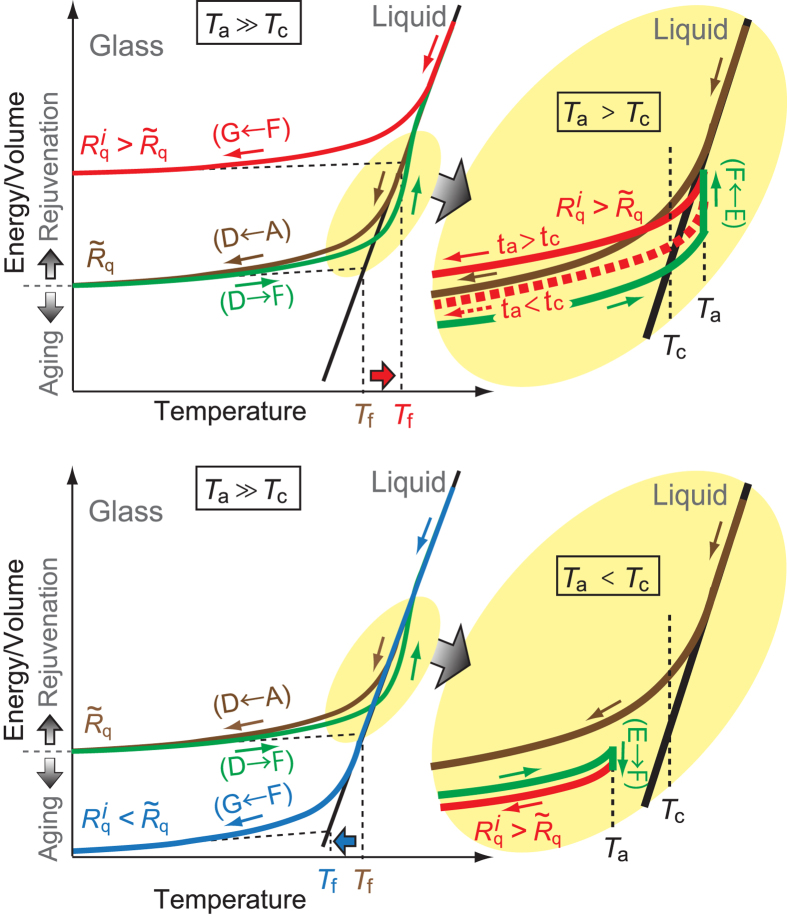
Schematic illustration of the energy/volume change during the initial melt-quenching (A
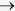
D) and subsequent thermal loading processes (D
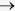
G) under conditions of 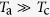 and 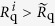 (top left); 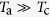 and 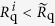 (bottom left); 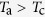 and 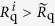 (top right); and 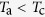 and 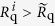 (bottom right). The equilibrium liquid is represented as a solid black line. In the top right figure, the solid red curve represents the energy/volume change under 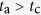 conditions, while the dotted red curve represents that under 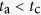 conditions. In the top and bottom left figures, the vertical dotted lines represent the fictive temperature 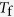, at which the extrapolated glass line intersects the equilibrium liquid line. The aging decreases and rejuvenation increases 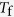 by changing the energy/volume versus temperature curve of the glass.

Figure 3 shows a schematic of the energy/volume change during the initial melt-quenching (A
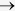
D) and subsequent thermal loading processes (D
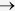
G). (1) If the annealing temperature 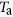 is much higher than 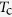 (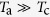), then an amorphous metal may quickly “relax” to an equilibrium liquid state within the heating stage (green curve in Fig. 3, top and bottom left) before the annealing process starts. As the equilibrium liquid is cooled (F
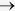
G), sooner or later the liquid deviates from the equilibrium liquid state and eventually the structure is frozen into a glass. A cooling rate 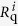 higher than 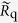 induces an earlier deviation from the equilibrium liquid and leads to a rejuvenated glass with a higher energy/volume (red curve in Fig. 3, top left), while a cooling rate lower than 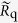 leads to an aged glass (blue curve in Fig. 3, bottom left). (2) If 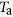 is slightly above 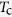 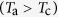, then the annealing time 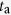 becomes a key factor in addition to the cooling rate 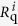 because the relaxation time to the equilibrium liquid is considerable compared with the heating and annealing times. Thus, during isothermal annealing the energy/volume increases to approach that of the equilibrium liquid. We actually observed the energy increase during the isothermal annealing process under 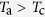 conditions (see Supplementary Information, Fig. S4). As shown in Fig. 3 (top right), a longer annealing time leads to a larger energy/volume increase during isothermal annealing and eventually to a rejuvenated glass after cooling (solid red curve), while a shorter annealing time may lead to an aged glass (dotted red curve). Therefore, there is a critical minimum annealing time for rejuvenation 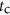; rejuvenation is realized only if 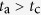. (3) If 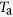 is below 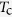 (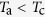), then the annealing-cooling process always leads to aged glass as shown in Fig. 3 (bottom right). In Fig. 3 (top and bottom left), the fictive temperature 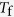 (Ref. 25) is also indicated, at which the extrapolated glass line intersects the equilibrium liquid line. In a similar manner to 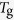, glass produced at a higher cooling rate from a high temperature liquid has a higher fictive temperature and vice versa^26^.

### Change in the topological order by rejuvenation

**Figure 4:**
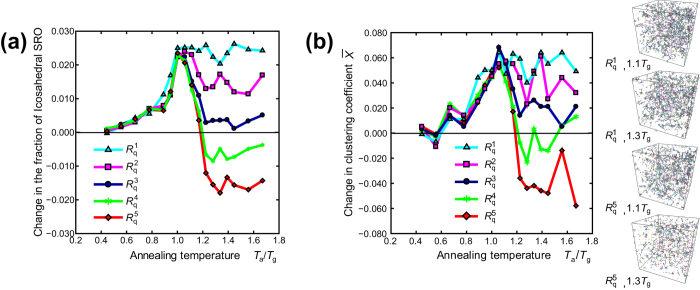
The change in the topological order induced by the thermal loading process. (**a**) Change in the fraction of the icosahedral atomic cluster through thermal loading with different final cooling rates 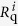. The as-quenched model has an icosahedral cluster fraction of 0.081. (**b**) Change in the average cluster coefficient of the icosahedral MRO 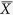 during the thermal loading process (D
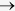
G) with different final cooling rates 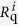. The 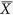 value of the as-quenched model is 0.327. The four figures to the right of the graph represent the MRO network structure^27^ of the annealed models. The final cooling rate and annealing temperature are shown at the bottom left of each figure.

We investigated the change in the topological order induced by the thermal loading process. The internal structures of amorphous metals, such as the short-range order (SRO) and medium-range order (MRO) have been attracted much attention, because they are correlated with the intrinsic properties of amorphous metals. Figure 4(a) shows the change in the fraction of icosahedral SRO, which has an energetically stable, high packing structure and is known to be a core amorphous metal structure. The figure shows clearly that aging increases and rejuvenation decreases icosahedral SRO.

Figure 4(b) shows the topological MRO composed of icosahedra. To characterize the MRO network structure, we employ the MRO clustering coefficient parameter 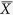, which was employed in our previous work^27^. For the center atom of the icosahedron 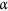, the clustering coefficient parameter 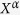 can be defined as, 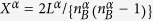, where 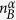 is the icosahedral bond number, and 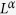 is the number of triangular geometries. As 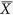 is the average clustering coefficient parameter over all icosahedra in an amorphous model, a large 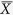 value implies a highly developed MRO network structure. As shown in Fig. 4(b), 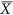 increases during the thermal loading process (D
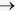
G) when the annealing temperature 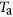 is below 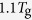 or the cooling rate is below 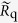. In contrast, although some fluctuation is observed, 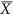 decreases when 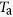 is above 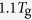 and the cooling rate is above 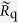.

Together with Fig. 2(a) and Fig. 4, we conclude that aging increases and rejuvenation decreases the topological SRO and MRO, which are typical characteristics of glass structures. The clear correspondence between the potential energy profile in Fig. 2(a) (and the volume profile in Supplementary Information, Fig. S3) and the SRO and MRO in Fig. 4 indicates that aging and rejuvenation change the macroscopic structural properties of amorphous metals, such as the free volume, by changing the internal topological order.

### Change in the mechanical properties by rejuvenation

**Figure 5:**
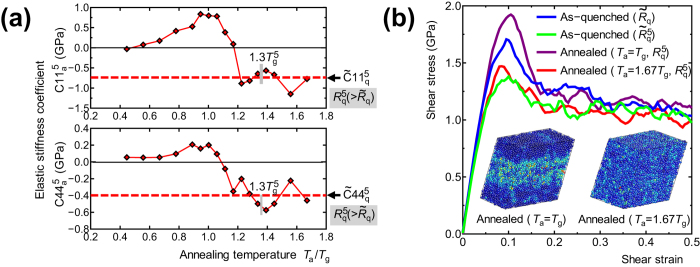
The change in the mechanical properties induced by the thermal loading process. (**a**) Change in the elastic stiffness coefficients 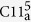 and 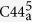 during thermal loading (process D
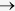
G) at a final cooling rate of 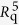 and various annealing temperatures 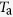. The elastic stiffness coefficients of the as-quenched model with a cooling rate 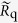 (state G) are 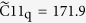 GPa and 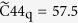 GPa; these are adopted as reference values. The elastic stiffness coefficient of the as-quenched model with a cooling rate 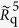 are shown by the dashed lines. (**b**) The shear stress change during the shear deformation tests for four models; two as-quenched models (state D) with different cooling rates, 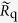 and 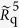 (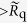), and two annealed models (state G) with different annealing temperatures, 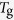 (aged model) and 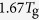 (rejuvenated model) with the same cooling rate of after annealing, 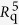
(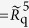). Inset figures represent snapshots of the two annealed models colored by local Mises strain at the same 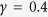 engineering shear strain.

To demonstrate the possibility of controlling the mechanical properties via aging and rejuvenation, the change in elastic stiffness due to thermal loading (process D
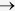
G) was investigated. The elastic stiffness coefficients of the as-quenched model with a cooling rate 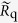 (state D in Fig. 1), 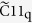 and 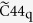, were calculated from the second spatial derivative of the total potential energy 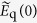; these 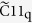 and 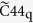 values are taken as the reference. The elastic stiffness coefficients of the models annealed at 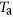 and with a final cooling rate 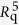 (state G) were also calculated, and the change in the elastic stiffness coefficients due to thermal loading (process D
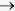
G), 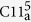 and 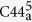, are shown Fig. 5(a). Both 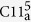 and 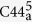 increase in the case of aging, while they decrease in the case of rejuvenation. Moreover, both values saturate in the high 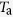 region and take values of 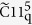 and 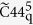, respectively, which are the elastic stiffness coefficients of the as-quenched model with a cooling rate of 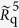 (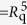). The clear correspondence between the potential energy profile (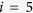) in Fig. 2 and the elastic stiffness coefficient profile in Fig. 5(a) shows the possibility of controlling the elastic properties of amorphous metals via thermally induced rejuvenation and aging. The correlation between SRO in Fig. 4(a) and elastic stiffness in Fig. 5(a) in this study agrees with that found in our previous studies^27^,^28^, where the elastic constant increases with increasing icosahedral SRO.

Plastic deformation behavior of the “aged model” and “rejuvenated model” was also investigated by simple shear MD deformation tests, in which the models were applied incremental affine shear strain at 0 K. At each strain step, the atomic structure and simulation box were relaxed using the conjugate gradient method. Figure 5(b) shows the shear stress change during the shear deformation tests for four models; two as-quenched models (state D) with different cooling rates, 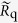 and 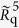
(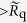), and two annealed models (state G) with different annealing temperatures, 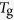 (aged model) and 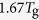 (rejuvenated model) with the same cooling rate of after annealing, 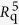
(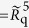). All the model shows a maximum shear stress at around 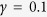 engineering shear strain, and the peak shear stress increases by the thermal loading process D
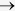
G with the annealing at 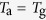 and decreases with the annealing at 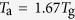. The two figures of atomic structure in Fig. 5(b) represent snapshots of the annealed models at 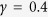 engineering shear strain, in which atoms are colored by Mises strain^29^. The aged model shows an inhomogeneous deformation, in which Mises strain is localized into band region parallel to shear direction, while the rejuvenated model shows more homogeneous deformation. The clear difference in deformation mode between the aged and rejuvenated models demonstrates that the aging and rejuvenation can change the plastic behavior of amorphous metals. It is well known that deformation at 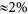 uniaxial strain of well-aged amorphous metals at room temperature is severely localized into narrow band region, so called shear banding, and the shear banding severely limits the macroscopic deformability of amorphous metals because of the local melting^30^,^31^,^32^,^33^. The homogeneous deformation in the rejuvenated model suggests that the rejuvenation conduces better macroscopic deformability to amorphous metals. Tensile ductility of amorphous metal induced by mechanical rejuvenation has been reported in a recent experimental study^34^.

We also experimentally observed the change in the mechanical properties via rejuvenation through a micro Vickers hardness test (see Supplementary Information, Fig. S5).

Recently, the critical fictive temperature concept 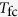 for plasticity in amorphous metals has been proposed^13^. The critical fictive temperature is a characteristic of amorphous metals: an amorphous metal is ductile in the case of 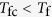 and brittle in the case of 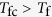. Since rejuvenation increases 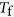 as schematically shown in Fig. 3, it would improve the ductility of amorphous metals through the prevention of catastrophic shear localization behavior such as shear banding. We actually demonstrated that rejuvenation enhances more homogenous deformation in Fig. 5(b), which supports a fictive-temperature-based discussion of ductility in amorphous metals.

## Conclusion

In summary, we have conducted thermal loading MD simulations of an already-formed amorphous metals subjected to heating, isothermal annealing, and second quenching and have produced a rejuvenation map with respect to the annealing temperature and second quenching rate that allows conditions to be found to control the level of rejuvenation. We found that thermal rejuvenation occurs via a thermal processing of isothermal annealing at temperatures above 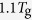 and subsequent quenching at a cooling rate that is higher than that of the initial quenching process. The level of rejuvenation increases with increasing annealing temperature and quenching rate. We also experimentally demonstrated control of the level of thermal rejuvenation in Zr_55_Al_10_Ni_5_Cu_30_ bulk glassy alloy. The above results are qualitatively consistent with analytical models of fictive temperature evolution in quenching and heating glass DSC analysis^26^, but now adding a constructive notion of applying the heating-quenching process *multiple* times to engineer glass properties, with quantitative proofs that the scheme actually works.

The fact that rejuvenation is realized via annealing even slightly above 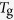 and subsequent quenching is important for modifying the glass properties after the material has been cast and processed into near component shape because amorphous metals are easy to handle at temperatures slightly above 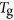 because of their high resistance to shape changes and/or crystallization.

One would have the flexibility of applying the thermal rejuvenation very locally, using for example transient laser heating; if the laser-heated spot is very small, the local cooling rate afterwards could be much higher than with the entire glassy block cooling down from melt. In practical thermal loading, the achieved level of rejuvenation will vary across the sample because heterogeneous heat transfer and heat conduction over time and space are expected. If we need to demonstrate the details of the heterogeneity in an actual heat treatment process, then we could employ a coarse-grained heat transfer and temperature distribution analysis such as FEM in combination with our rejuvenation map. This analysis framework could be a useful engineering tool for predicting heterogeneous rejuvenation and engineering the desired rejuvenation distribution while avoiding crystallization and/or gross shape changes with certain boundary conditions. Recently, nanoimprinting of metallic glass^35^ has been developed using a thermoplastic forming process slightly above 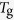, which allows the realization of low-cost fabrication of micro- and nanodevices. Our study proposes that a thermoplastic forming process consisting of annealing slightly above 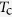 and then rapid cooling before the glass relaxes into equilibrium liquid would avoid embrittlement by aging in the thermoplastic forming process.

## Additional Information

**How to cite this article**: Wakeda, M. *et al.* Controlled Rejuvenation of Amorphous Metals with Thermal Processing. *Sci. Rep.* **5**, 10545; doi: 10.1038/srep10545 (2015).

## Supplementary Material

Supplementary Information
