# Supplementary material for: Controlled Rejuvenation of Amorphous Metals with Thermal Processing
Source: Sci Rep. 2015 May 26;5:10545. doi: 10.1038/srep10545 (PMC4443766; doi:10.1038/srep10545)
Supplement: Supplementary Information [file srep10545-s1.pdf]

Supplementary Information

Controlled Rejuvenation of Amorphous Metals with Thermal Processing

Masato Wakeda,[1,*] Junji Saida,[2] Ju Li,[3] and Shigenobu Ogata[1,4,†]

[1]*Graduate School of Engineering Science, Osaka University,*
1-3 Machikaneyama, Toyonaka, Osaka, 560-8531, Japan

[2]*Frontier Research Institute for Interdisciplinary Sciences,*
Tohoku University, Aramaki aza Aoba 6-3,
Aoba-ku, Sendai, Miyagi, 980-8578, Japan

[3]*Department of Nuclear Science and Engineering and*
Department of Materials Science and Engineering,
Massachusetts Institute of Technology, 77 Massachusetts Avenue,
Cambridge, Massachusetts, 02139, USA

[4]*Center for Elements Strategy Initiative for Structural Materials (ESISM),*
Yoshida Honmachi, Sakyo-ku, Kyoto University, Kyoto, 606-8501, Japan

[*] wakeda@me.es.osaka-u.ac.jp
[†] ogata@me.es.osaka-u.ac.jp

I. ESTIMATION OF THE GLASS TRANSITION TEMPERATURE T_g

The glass transition temperatures T_g^i are estimated from the temperature of the kink point in the volume-temperature curves of the melt-quenching process with cooling rates of $\widetilde{R}_q^i$, as shown in Fig. S1. The estimated T_g^i values of $Cu_{50}Zr_{50}$ model are 860, 886, 898, 924, and 941 K for $i = 1 - 5$, respectively. As seen in the enlarged view in the inset in Fig. S1, a slower cooling rate leads to a smaller volume (i.e., a smaller free volume).

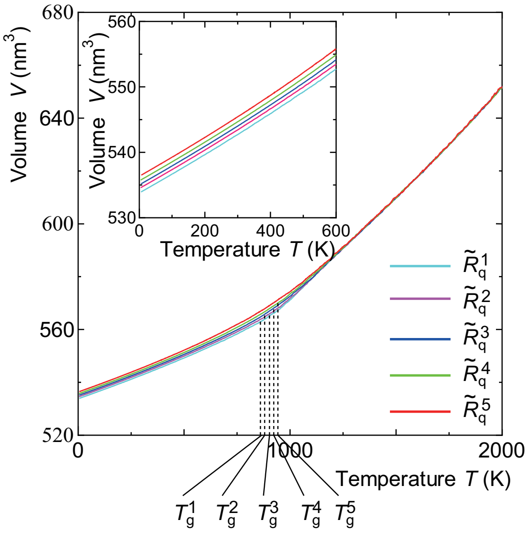

Figure S1. Volume as a function of temperature for the melt-quenching process with a cooling rate $\widetilde{R}_q^i$. The glass transition temperatures T_g^i are denoted by the vertical dashed lines. The inset shows the volume-temperature curves in a temperature range of 0 to 600 K.

II. DEPENDENCIES OF THE ANNEALING TIME AND ATOMIC COMPOSITION

We simulated the D→G thermal loading process with different annealing times (t_a = 500 and 1000 ps) for different atomic compositions ($Cu_{30}Zr_{70}$ and $Cu_{57}Zr_{43}$). Figure S2 shows the generated excess potential energy $E_a^i(0)$ ($i = 5$ only is shown for simplicity) before and after the thermal loading for t_a = 500, 1000, and 2000 ps and (a) $Cu_{30}Zr_{70}$, (b) $Cu_{50}Zr_{50}$, and (c) $Cu_{57}Zr_{43}$. In all cases, rejuvenation is realized when the cooling rate after annealing is higher than that of the initial melt-quenching process, i.e., $R_q^i > \widetilde{R}_q$, and the annealing temperature is higher than the critical temperature, i.e., $T_a > T_c$, where T_c is roughly $1.1T_g$. This result is consistent with our conclusion derived from the results in Fig. 2(a) in the main text. For all compositions, the level of aging

increases with increasing annealing time, while the level of rejuvenation is independent of the annealing time. Although aging requires a significant amount of time to reach a thermodynamically more stable state, erasing the aging history can be easily achieved by thermal activation even on an MD time scale.

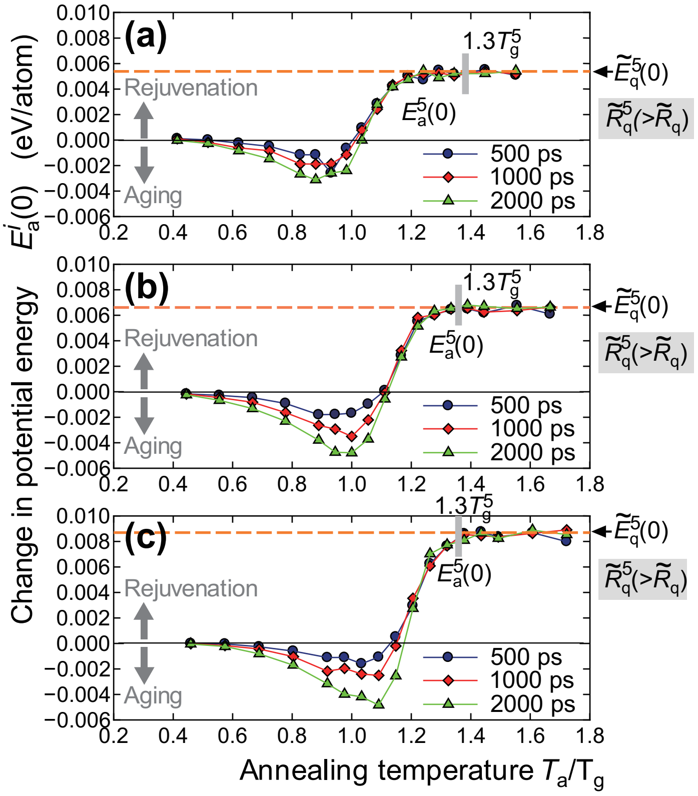

Figure S2. Change in the potential energy via subsequent thermal loading for three different annealing times t_a = 500, 1000, and 2000 ps and the (a) $Cu_{30}Zr_{70}$, (b) $Cu_{50}Zr_{50}$, and (c) $Cu_{57}Zr_{43}$ amorphous models. $E_q^5(0)$ is the potential energy of the annealed model with a cooling rate of R_q^5, while $\widetilde{E}_q^5(0)$ shown by the horizontal dashed line is the potential energy of the as-quenched model with a cooling rate of $\widetilde{R}_q^5$ (see Fig. 1 in the main text). The annealing temperature T_a is normalized by the glass transition temperature T_g in the melt-quenching process of each atomic composition with a cooling rate $\widetilde{R}_q$; in (a), (b), and (c), T_g = 967, 898, and 871 K, respectively.

III. CHANGE IN VOLUME CAUSED BY THE THERMAL LOADING PROCESS (D→G)

Figure 2(a) in the main text revealed the potential energy difference before and after thermal loading. The aging and rejuvenation levels are evaluated in a similar manner based on the volumes,

$V_a^i(0)$, as shown in Fig. S3, where $V_a^i(0)$ are the volumes of the annealed models with cooling rates R_q^i. Since the rejuvenation increases the free volume (while aging decreases the free volume), the excess volume can also be defined as the " rejuvenation level " if it is positive and as the " aging level " if it is negative. Here, $\widetilde{V}_q(0)$ is the volume of the as-quenched model with a cooling rate $\widetilde{R}_q$, which we set as a reference so that each volume in Fig. S3 implicitly represents an increment from $\widetilde{V}_q(0)$. The volume curves show qualitatively the same profile, including the critical temperature T_c, as the potential energy curves in Fig. 2(a) in the main text.

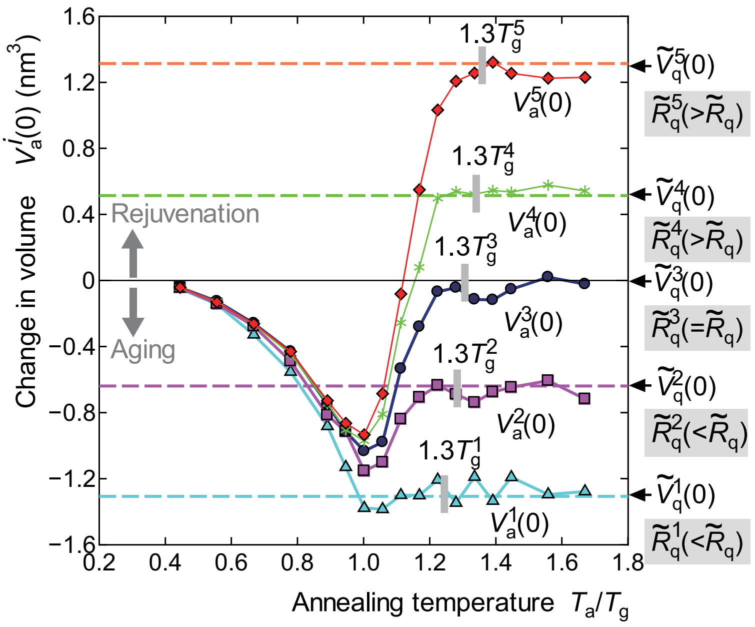

Figure S3. Change in the volume caused by the thermal loading process. The dashed lines represent the $\widetilde{V}_q^i(0)$ values, and $V_a^i(0)$ and $\widetilde{V}_q^i(0)$ are the volume of the annealed model with a cooling rate R_q^i and the volume of the as-quenched model with a cooling rate $\widetilde{R}_q^i$ $(= R_q^i)$, respectively.

IV. CHANGE IN THE POTENTIAL ENERGY DUE TO REHEATING AND ANNEALING

Here, we shows the potential energies $\widetilde{E}_q(T_a)$, $E_h(T_a)$, and $E_a(T_a)$, which represent the instantaneous potential energy at $T = T_a$ in the initial melt-quenching process (state C), and the potential energies just before and after annealing at T_a (states E and F), respectively, in Fig. S4(a). The instantaneous potential energies at finite temperatures were computed by taking a simple average of the total potential energies over 10^4 successive MD time steps (= 10 ps), while the potential energies at 0 K were obtained from a single snapshot of the frozen amorphous model for which

the potential energy was minimized via the conjugate gradient method.

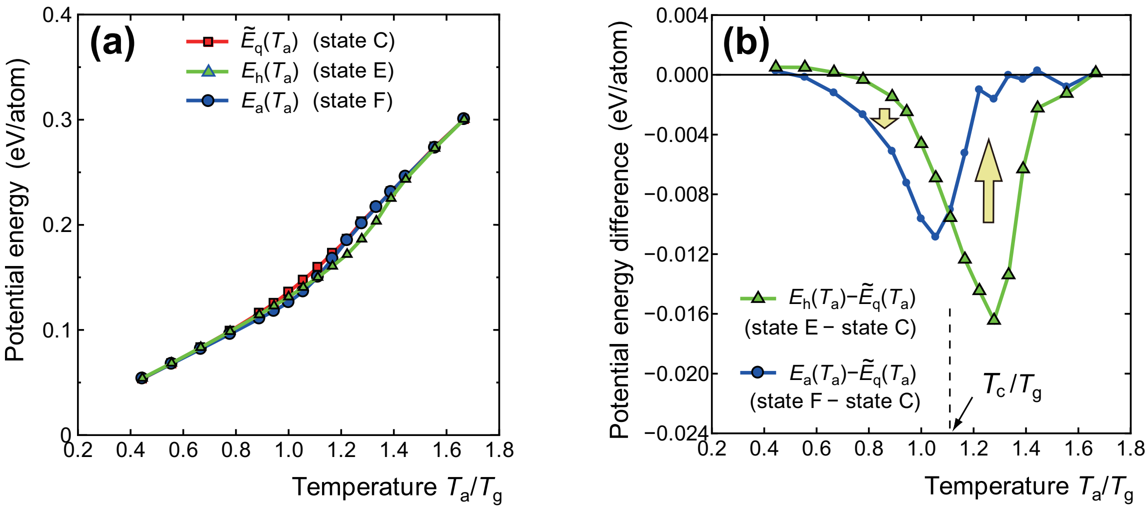

Figure S4. Change in the potential energy due to quenching, reheating, and annealing. (a) The instantaneous potential energy in the initial melt-quenching process at temperatures T_a (state C) and the potential energies before and after the isothermal annealing process (states E and F, respectively) are denoted by $\widetilde{E}_q(T_a)$, $E_h(T_a)$, and $E_a(T_a)$, respectively. (b) The potential energy differences $E_h(T_a) - \widetilde{E}_q(T_a)$ and $E_a(T_a) - \widetilde{E}_q(T_a)$ as a function of temperature. The vertical dotted line represents the critical temperature T_c. If the annealing temperature is higher than T_c, isothermal annealing (E→F) increases the potential energy.

Figure S4(b) shows the energy differences $E_h(T_a) - \widetilde{E}_q(T_a)$ and $E_a(T_a) - \widetilde{E}_q(T_a)$ as a function of temperature. Note that in this work we always take the potential energy of the as-quenched model $\widetilde{E}_q(0)$ as the energy reference so that potential energy values presented here are always defined relative to $\widetilde{E}_q(0)$. The temperature is normalized by the glass transition temperature T_g for a cooling rate $\widetilde{R}_q$. We see from Fig. S4(b) that the absolute values of the potential energy differences, $|E_h(T_a)-\widetilde{E}_q(T_a)|$ and $|E_a(T_a)-\widetilde{E}_q(T_a)|$, increase with increasing annealing temperature T_a up to $T_a \approx 1.3T_g$ in the case of $E_h(T_a)-\widetilde{E}_q(T_a)$ and $T_a \approx T_g$ in the case of $E_a(T_a)-\widetilde{E}_q(T_a)$. A negative potential energy difference indicates that the models just before and after isothermal annealing at T_a (states E and F in Fig. 1 in the main text) are more stable than the instantaneous liquid or amorphous state in the initial melt-quenching process at T_a (state C). The difference between $E_h(T_a) - \widetilde{E}_q(T_a)$ and $E_a(T_a)-\widetilde{E}_q(T_a)$, however, represents the change in the potential energy during isothermal annealing (E→F). If the annealing temperature is lower than $\approx T_c$, then the amorphous state moves toward a lower free-energy configuration via atomic structure rearrangement during the annealing process

because in principle any quenched glasses have not been fully aged owing to the finite cooling rate of the quenching process when it was made. Therefore, as is well known, annealing below T_g ($< T_c$) generally induces further aging[1], as seen in Fig. S4(b). In contrast, the value of $|E_a(T_a) - \widetilde{E}_q(T_a)|$ for annealing at $T_a > T_c$ decreases with increasing annealing temperature, falling to zero at $T_a \approx 1.3T_g$, which suggests that the amorphous state just after isothermal annealing (state F) is equivalent to the instantaneous liquid or amorphous state of the initial quenching process at the same temperature (T_a; state C). This demonstrates that the aging history during the process C→D in Fig. 1 in the main text is completely erased by the heating and isothermal annealing processes above $1.3T_g$ (D→E→F) due to the large thermal activation and full recovery of ergodicity, but the aging history is only partially erased by annealing below $1.3T_g$. These results are qualitatively consistent with previous analytical model of fictive temperature evolution during glass differential scanning calorimetry (DSC) analysis[2].

V. CHANGE IN MICRO VICKERS HARDNESS INDUCED BY THE EXPERIMENTAL ANNEALING AND SUBSEQUENT QUENCHING PROCESS

$Zr_{55}Al_{10}Ni_5Cu_{30}$ data in Fig. 2(b) in the main text experimentally demonstrates the realization of thermal rejuvenation via annealing and subsequent quenching process. For the experimental $Zr_{55}Al_{10}Ni_5Cu_{30}$ bulk glassy alloy, we also evaluated change in a micro Vickers hardness between fully relaxed state and annealed state (detailed explanation about the two states are mentioned in the main text)[3]. As seen in Fig. S5, the change in Vickers hardness is negative and decreases with increasing cooling rate from -10±11 at $R_{\mathrm{Exp.}}$=0.33 K/s to -25±10 at $R_{\mathrm{Exp.}}$=4.4 K/s, showing a clear effect of thermal rejuvenation on the mechanical properties.

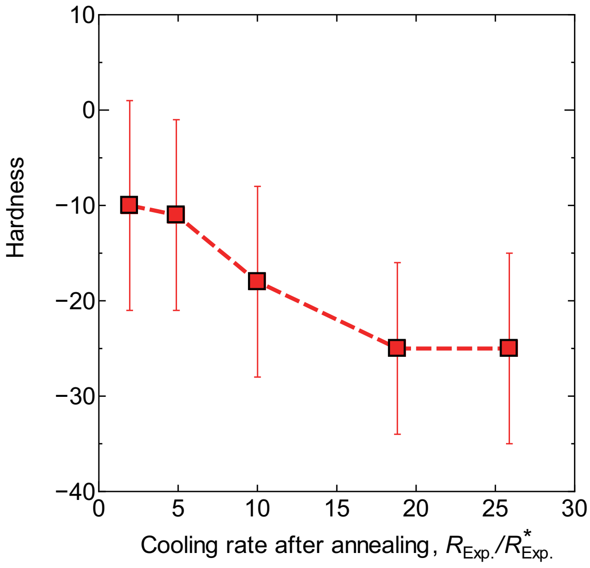

Figure S5. Change in micro Vickers hardness via the experimental thermal loading process. For each sample, micro Vickers hardness tests with a load of 20 g were conducted at least 10 measurements. The Vickers hardness of the fully relaxed alloy is 475-15.

VI. COOLING RATE INFLUENCED TEMPERATURE RANGE

All the curves in Fig. 2(a) in the main text show plateau behavior with respect to T_{a} above $1.3T^{i}_{\mathrm{g}}$, where the $1.3T^{i}_{\mathrm{g}}$ values are shown by the vertical bars. The plateau values are equivalent to the $\widetilde{E}^{i}_{\mathrm{q}}(0)$ values shown by the dashed line, which were obtained by performing melt-quenching from 3000 to 0 K with cooling rates of $\widetilde{R}^{i}_{\mathrm{q}}(= R^{i}_{\mathrm{q}})$. It is worth mentioning that these $1.3T^{i}_{\mathrm{g}}$ temperatures are not sensitive to the annealing time t_{a} (see Fig. S2). We can postulate from the consistency in the case of $T_{\mathrm{a}} > 1.3T^{i}_{\mathrm{g}}$ that the aging history in the amorphous state during the initial quenching process (C→D) was completely erased by the thermal loading process (D→F), as discussed in relation to Fig. S4. In other words, the amorphous states at C and F are thermodynamically equivalent, as statistical ergodicity is achieved even with nanosecond timescale. Therefore, in the case of $T_{\mathrm{a}} > 1.3T^{i}_{\mathrm{g}}$, if a cooling rate during the second quenching process (F→G) than that of the initial quenching process (C→D), i.e., $R^{i}_{\mathrm{q}} > \widetilde{R}_{\mathrm{q}}$, is used, then thermal rejuvenation is inevitably realized because less aging occurs during the second quenching process. In addition, the consistency of the potential energies $E^{i}_{\mathrm{a}}(0)$ and $\widetilde{E}^{i}_{\mathrm{q}}(0)$ also provides some important insight into the cooling-rate-influenced temperature range. Since amorphous states at C and F are thermody-

namically equivalent, it is possible to regard the annealed model with $E_{\mathrm{a}}^{i}(0)$ as having quenching histories with a cooling rate of $\widetilde{R}_{\mathrm{q}}$ from 3000 K to T_{a} and a cooling rate of R_{q}^{i} from T_{a} to 0 K. In contrast, the as-quenched model of potential energy $\widetilde{E}_{\mathrm{q}}^{i}(0)$ undergoes the quenching process with a constant cooling rate of $\widetilde{R}_{\mathrm{q}}^{i}(= R_{\mathrm{q}}^{i})$ from 3000 K to 0 K. Therefore, the potential energy consistency implies that the cooling rate above T_{a} $(> 1.3T_{\mathrm{g}}^{i})$ does not affect the constructed amorphous state.

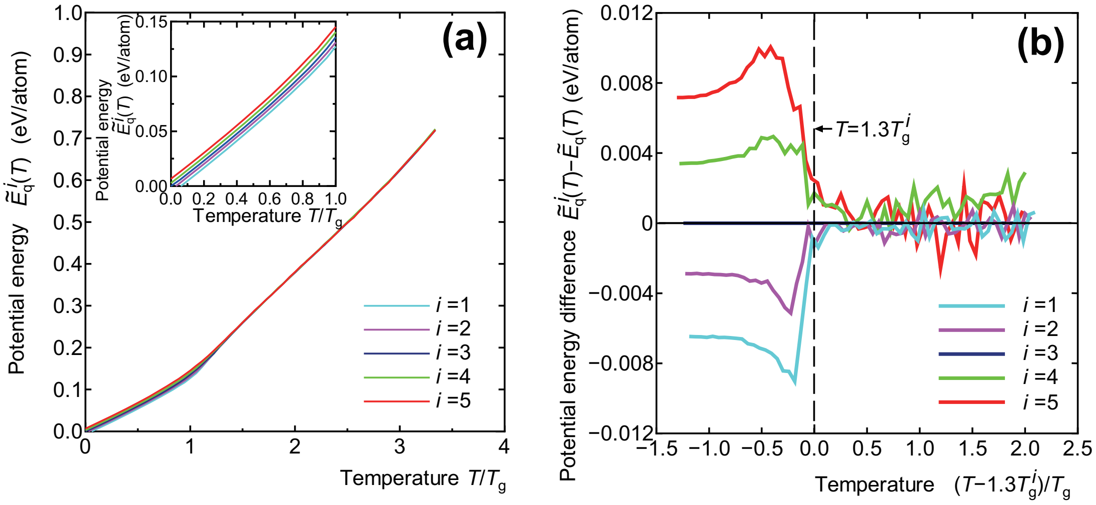

Figure S6. Potential energy change during the melt-quenching process with various cooling rates. (a) Change in the potential energy $\widetilde{E}_{\mathrm{q}}^{i}(T)$ in the melt-quenching process (B→D) at temperature T for five different cooling rates$\widetilde{R}_{\mathrm{q}}^{i}$ ($i = 1 - 5$). (b) The potential energy difference, $\widetilde{E}_{\mathrm{q}}^{i}(T) - \widetilde{E}_{\mathrm{q}}(T)$, where $\widetilde{E}_{\mathrm{q}}(T)$ $(= \widetilde{E}_{\mathrm{q}}^{3}(T))$ is the potential energy of the melt-quenching process at T with a cooling rate $\widetilde{R}_{\mathrm{q}}(= \widetilde{R}_{\mathrm{q}}^{3})$.

The change in the potential energy during the five different melt-quenching processes is shown in Fig. S6(a). The differences between the instantaneous potential energies at each temperature T, $\widetilde{E}_{\mathrm{q}}^{i}(T) - \widetilde{E}_{\mathrm{q}}(T)$, where $\widetilde{E}_{\mathrm{q}}(T)(= \widetilde{E}_{\mathrm{q}}^{3}(T))$ is the potential energy of the initial quenching process, are also shown in Fig. S6(b), where the temperature is given as $(T - 1.3T_{\mathrm{g}}^{i})/T_{\mathrm{g}}$ to align the different $1.3T_{\mathrm{g}}^{i}$ values at the origin. The potential energy differences at high temperature are approximately zero, which means that the instantaneous state is independent of the cooling rate, i.e. statistical ergodicity is fully achieved even at nanosecond (MD) timescale. At temperatures below $1.3T_{\mathrm{g}}^{i}$, however, there is a significant dependence on the cooling rate: a slower cooling rate leads to a lower potential energy. Thus, above $1.3T_{\mathrm{g}}^{i}$, the instantaneous temperature dictates the current liquid or amorphous state, while below $1.3T_{\mathrm{g}}^{i}$ it is not only the instantaneous temperature but also the thermal history that influences the current liquid or amorphous state. Although there have been a few experimental studies of the temperature range, wherein the cooling rate affects the constructed

amorphous metals, Saida et al.[4, 5] have indicated that the cooling rate for temperatures below T_g + 160 K (≈ 1.23T_g) are dominant for the aging level and mechanical properties in Zr-based multicomponent metallic glasses. Our results are consistent with this conclusion.

[1] Egami, T. Structural relaxation in metallic glasses. *Ann. N.Y. Acad. Sci.* **371**, 238-251 (1981).

[2] Moynihan, C. T., Easteal, A. J. & Debolt, M. A., Tucker, J. Dependence of the Fictive Temperature of Glass on Cooling Rate. *J. Am. Ceram. Soc.* **59**, 12-16 (1976).

[3] Saida, J., Yamada, R. & Wakeda, M. Recovery of less relaxed state in Zr-Al-Ni-Cu bulk metallic glass annealed above glass transition temperature. *Appl. Phys. Lett.* **103**, 221910 (2013).

[4] Saida, J., Setyawan, A. D., Kato, H.& Inoue, A. Cooling Process and Cast Structure of Zr-Al-Ni-Cu-Based Bulk Metallic Glasses Produced in Various Atmospheres. *Metall. Mater. Trans. A* **42**, 1450-1455 (2011).

[5] Saida, J., Setyawan, A. D., & Matsubara, E. Effect of relaxation state on nucleation and grain growth of nanoscale quasicrystal in Zr-based bulk metallic glasses prepared under various cooling rates. *Appl. Phys. Lett.* **99**, 061903 (2011).
